# Engineering Breast Cancer Cells and hUMSCs Microenvironment in 2D and 3D Scaffolds: A Mechanical Study Approach of Stem Cells in Anticancer Therapy

**DOI:** 10.3390/bioengineering8110189

**Published:** 2021-11-20

**Authors:** Despoina Nektaria Metsiou, Foteini K. Kozaniti, Despina D. Deligianni

**Affiliations:** Laboratory of Biomechanics and Biomedical Engineering, Department of Mechanical Engineering and Aeronautics, University of Patras, Rion, 26504 Patra, Greece; fkozaniti@upatras.gr (F.K.K.); deliyian@upatras.gr (D.D.D.)

**Keywords:** cancer, stem cells, therapy, biomechanics, dynamic cell culture, bioreactor

## Abstract

Cell biomechanics plays a major role as a promising biomarker for early cancer diagnosis and prognosis. In the present study, alterations in modulus of elasticity, cell membrane roughness, and migratory potential of MCF-7 (ER+) and SKBR-3 (HER2+) cancer cells were elucidated prior to and post treatment with conditioned medium from human umbilical mesenchymal stem cells (hUMSCs-CM) during static and dynamic cell culture. Moreover, the therapeutic potency of hUMSCs-CM on cancer cell’s viability, migratory potential, and F-actin quantified intensity was addressed in 2D surfaces and 3D scaffolds. Interestingly, alterations in ER+ cancer cells showed a positive effect of treatment upon limiting cell viability, motility, and potential for migration. Moreover, increased post treatment cell stiffness indicated rigid cancer cells with confined cell movement and cytoskeletal alterations with restricted lamellipodia formation, which enhanced these results. On the contrary, the cell viability and the migratory potential were not confined post treatment with hUMSCs-CM on HER2+ cells, possibly due to their intrinsic aggressiveness. The increased post treatment cell viability and the decreased cell stiffness indicated an increased potency for cell movement. Hence, the therapy had no efficacy on HER2+ cells.

## 1. Introduction

Breast cancer is the leading cause of cancer-related morbidity and mortality in postmenopausal women [1]. Although prognosis has improved due to advances in diagnostic and surgical techniques, breast cancer remains one of the most challenging diseases to treat [2]. However, the side effects of anti-cancer chemotherapy remain a major source of concern despite the improved efficacy and enhanced survival offered by modern treatments [3]. Nowadays, tumor-targeted drug delivery has the potential to improve therapeutic efficacy and mitigate the non-specific toxicity of anticancer drugs [4]. Nevertheless, the short half-life of most chemotherapeutic drugs and the high systemic toxicity inhibits the effective delivery of anti-cancer drugs to the tumor [5]. Stem cells are promising as a regenerative anti-cancer cell therapy [6]. Previous studies have shown that stem cells, and more precisely, human umbilical mesenchymal stem cells (hUMSCs), can attenuate tumor growth of triple-negative cancer and bronchioloalveolar carcinoma [7,8].

Overall, the use of hUCMSCs in cancer therapy outlines a plethora of attractive advantages, including a non-invasive collection procedure, low risk of infection, non-tumorigenicity, and low immunogenicity [9]. Moreover, the latest studies have shown that secreted proteins from stem cells may attenuate tumor growth and inhibit apoptosis [10,11].

However, whether the clinical use of hUMSCs is an optimal choice as anti-cancer therapy is not yet determined, and the necessity of further research is imperative to establish the therapeutic effect of stem cells on tumor growth. Nevertheless, the era of use of stem cell therapy has arrived and it is a fruitful field of investigation [12].

This study attempted to bring forward and introduce the novel therapeutic opportunities of using culture medium from stem cells in ER+ and HER2+ breast cancer cells in a mechanical perspective. The purpose of this study was to investigate how the conditioned medium (CM) from hUMSCs could possibly affect breast cancer cells with different molecular subtypes. How could it possibly alter the post treatment cell viability, the morphology, and biomechanics of breast cancer cells, namely elastic Young’s modulus (cell stiffness) and membrane roughness, and how these alterations could affect the metastatic potential of breast cancer cells. Particularly, we evaluated the effect of hUMSCs-CM as a therapy on breast cancer cells, depending on the static and dynamic culture microenvironment. Initially, we used MCF-7 cells, which express estrogen receptors and are categorized as ER+, and secondly, we used SKBR-3 cells, which overexpress human epidermal growth factor receptor 2 with HER2+ immunoprofile [13,14].

More specifically, this work was conducted to model a dynamic culture microenvironment via a bioreactor and two-dimensional (2D) surfaces or three-dimensional (3D) scaffolds in order to satisfactorily simulate in vitro homeostasis. 3D polycaprolactone (PCL) scaffolds were fabricated via electrospinning and the cells were seeded to create a 3D microenvironment. The morphology and porosity of 3D scaffolds were assessed with SEM. Cells were proliferated into a static incubator or into the bioreactor in order to exhibit dynamic culture. Cell viability was assessed via the MTT method, and the elasticity of cells was determined via micropipette aspiration technique, while the cell membrane roughness through atomic force microscopy (AFM). Accordingly, the cell membrane morphology was studied with confocal microscopy and the mean fluorescence intensity of cytoskeletal F-actin was evaluated. Finally, the potential for cell migration was estimated via wound healing assay. The aforementioned methods were implemented prior to and post treatment with hUMSCs-CM for 24 h and 48 h, respectively.

## 2. Materials and Methods

### 2.1. Cell Culture and Reagents

hUMSCs donated to our study from the Hellenic Cord Blood Bank of the Biomedical Research Foundation of the Academy of Athens. hUMSCs were isolated from the Wharton’s jelly of umbilical cords according to the protocol which analytically has been described in the study of Chatzistamatiou et al. [15] complied to ethical standards and local ethics committee. Adenocarcinoma cell lines SKBR-3 and MCF-7 purchased from ATCC (USA) were cultured as in previous studies [16]. Briefly, DMEM supplemented with 2 mM L-glutamine was used as a culture medium. Both cell lines were supplemented with 100 μg/mL penicillin G/streptomycin, 50 μg/mL gentamycin, and 10% fetal bovine serum (FBS). Static culture cells were incubated at 37 °C, 5% CO_2,_ and 100% humidity. Cancer cells exhibited dynamic culture into the bioreactor at steady temperature 37 °C and 5% CO_2_ conditions but with a constant flow of culture medium.

### 2.2. Preparation of 2D Surfaces

Regarding 2D surfaces, collagen-coated glass coverslips, with a range of 1.2–1.8 cm diameter, were used as 2D surfaces in this study. Specifically, Collagen Type I solution (50202 IBIDI) diluted at final concentration 20 μg/mL using 17.mM (0.1%) acetic acid. Before cell seeding, collagen-coated surfaces were incubated at approximately 30 min.

### 2.3. Fabrication of 3D Scaffolds

Polycaprolactone (PCL) pellets with a molecular weight of 80,000 g/mol and glacial acetic acid (purity 99.8%) were purchased from Sigma-Aldrich (St. Louis, MO, USA). All other chemicals were of reagent grade. 20% w/v solution of PCL in glacial acetic acid was prepared by mixing in a roller overnight. Gentle heating (40 °C) was used to help the dissolution of the polymer. The voltage in the electrospinning apparatus was set at 20 kV and the flow rate was 1 mL of solution per hour. Experiments were performed in ambient conditions, where the temperature and humidity were at the range of 20–25 °C and 40–50% RH, respectively. For further cell culture experiments, the electrospinning film was cut in a round shape with a 1.5 cm scaffold diameter, to fit into wells of 24-well culture plates. Before testing, scaffolds were washed in PBS for 24 h and sterilized with 70% ethanol solution for 2 h and a further 30 min of UV exposure. Finally, before cell seeding, PCL scaffolds were immersed in collagen Type I as described before and incubated at approximately 30 min.

### 2.4. Dynamic Cell Culture

**Figure 1:**
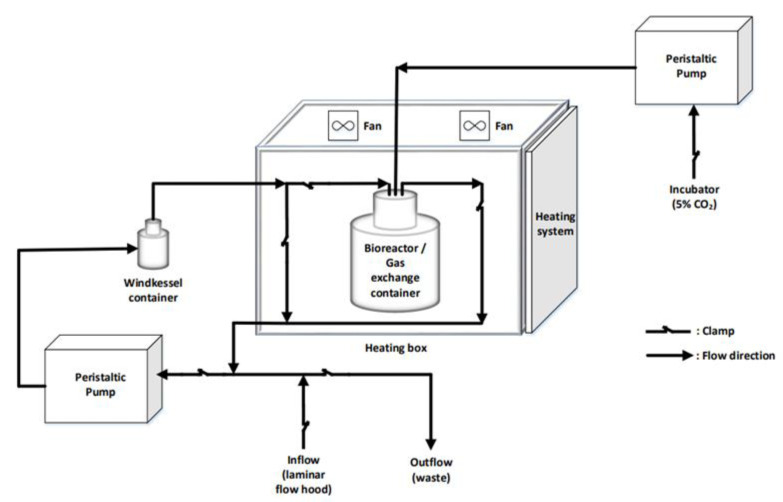
Conceptual view of the experimental setup of the bioreactor system to obtain dynamic cell culture.

A custom-made bioreactor was employed in order to achieve the dynamic cell culture, as depicted in Figure 1. Petri dish chambers were modified in a way that the culture medium could pass through the peristaltic pump into the Windkessel chamber, thus ensuring a smooth fluid flow, and then continue into the petri-dish chamber, where the cells were cultured in 2D coverslips or 3D PCL scaffolds. One peristaltic pump (Masterflex© 2021-12, Merck, NJ, USA) ensured the steady flow of culture medium, while another peristaltic pump (Masterflex© 2021-25, Merck, NJ, USA) ensured the constant 5% of CO_2_ level. The flow rate of the first pump was set at 1.7 mL/min (or 7 rpm with 4.3 mm diameter). The system was filled with culture medium via a by-pass network that started from a funnel, passed through the pump, the Windkessel chamber and the petri-dish chamber until the total system was filled with the adequate quantity of medium. In a similar way, the waste was discarded from the system in the disposal container at the end of the experiment. The diversion of the flow to the desired tubes was achieved with clamps or 3-way valves (LABOPLAST^®^). The tube was from highly quality pumps to withstand the loads (Masterflex© 20210-24). The Windkessel chamber was manually constructed. The temperature was controlled at 37 °C via a custom-made electronic system. It should be mentioned that the system was sterilized meticulously before and after the experimental set up; the current apparatus was washed with 70% ethanol, followed by UV exposure and distilled water. Phosphate-buffered saline buffer was finally used before the culture medium was inserted.

### 2.5. Treatment of Breast Cancer Cells with hUMSCSs-CM

hUCMSCs were cultured in Dulbecco’s modified Eagle’s medium (PanBiotech, Aidenbach, Germany) supplemented with 10% FBS to 90–95% confluence. Afterwards, the culture medium was harvested and filtered through 0.45 μm pore sterile filters (Lab Logistic Company) into aliquots, and stored at −80 °C. The hUMSCSs-CM were replaced every 24 h, in order to hinder the attenuation of bioactive substances [10]. Control breast cancer cells were cultured in Dulbecco’s modified Eagle’s medium.

### 2.6. Cell Viability and Cell Proliferation Assays

The MTT reduction assay was used to investigate the viability of cancer cells for different days of culture. A 5 mg/mL solution of MTT (3-(4,5-dimethylthiazol-2yl)-2,5-diphenyl-2H-tetrazoliumbromide; Sigma) was diluted 1:10 in serum-free medium. Scaffolds were washed with serum-free medium and incubated with MTT solution for 3 h at 37 °C. Then, the MTT solution was removed and DMSO was used to dissolve the formazan crystals. From each sample, 100 μL were added to 96-well plates in triplicate and the absorbance was read at 570 nm with a microplate reader (TECAN F200). The absorbance at 590 nm was used as a reference.

### 2.7. Migration Assay

Collective cell migration was examined in both static and dynamic conditions, using 2D scratch-wound assay. Cells were seeded in 6-well plates at a density of 2.5 × 10^5^ cells/well. After reaching 100% confluency, a scratch-wound was created and then cells were rinsed with PBS to remove dead cells. Culture medium with 10% FBS was added in the case of control cells and supplemented with hUMSCs-CM in treated cells, respectively. Cytosine b-D-arabinofuranoside hydrochloride 10 μM (Sigma-Aldrich Chemie Gmbh, Schnelldorf, Germany) was supplemented to avoid cell proliferation that affects migration. Regarding dynamic cell culture, 6-well plates were modified before inserting into the bioreactor. After 24 h and 48 h incubation, images of migrated cells were captured using an inverted microscope. After exporting images, the healing capacity was estimated as the percentage of migrated cell area.

### 2.8. Mechanical Properties of Breast Cancer Cells Prior to and Post Treatment

#### 2.8.1. Tensile Strength of PCL Scaffolds

Uniaxial tensile mechanical tests were performed until failure at fracture, using a Minimat (Rheometric Scientific, New Castle, DE, USA) testing device. According to ASTM D 882-02 Standard, 30 mm × 7.5 mm specimens were prepared and tested with a span of 11 mm long and a strain rate of 5 mm/min. The mean Young’s modulus of elasticity was computed from the linear region of the stress-strain curve. Ultimate stress at failure was also determined.

#### 2.8.2. Cell Elasticity

In this study, we investigated the elastic Young’s modulus of both MCF-7 and SKBR-3 breast cancer cells prior to and post treatment with hUMSCs-CM at different time points. A micropipette aspiration technique was used to determine the elastic Young’s modulus. More specifically, only the cell membrane was aspirated, thus avoiding nucleus aspiration, in order to obtain pure measurements of the cell membrane for the applied negative pressure at each time point. The experimental setup, as described before [16,17], consists of a borosilicate glass capillary micropipette, controlled by a mechanical micromanipulator, an inverted microscope with a camera, an adjustable fluid reservoir for creating fluid pressure in the micropipette, a pressure transducer, a monitor for digital display and a software. A range of pressure ΔP from 0.05 to 340 Pa was applied, to obtain a linear expression of cell deformation vs. aspiration pressure. When the micropipette radius was very small compared to the local radius of the cell surface, the projection of cell length, L, into micropipette, was predicted to be proportional to the aspiration pressure ΔP. Therefore, cancer cell elasticity was determined through the slope of the curve:

(1) $$\Delta P=f\left(\frac{L}{Dp}\right)$$

via the interpreted equation:

(2) $$E=3\varphi_{p}\;\left(R_{p}\frac{D_{p}}{2\pi L}\right)$$

where all variables have been thoroughly described in our previous work [16]. Briefly, E is the elastic Young’s modulus, $D_{p}$ the inner diameter of micropipette $R_{p}$ the inner pipette radius and $\Phi_{p}$ represents a function of the ratio of the pipette wall thickness to the pipette radius ($\Phi_{p\;}$= 2.0−2.1 when the ratio of the pipette wall thickness to radius is equal to 0.2–1.0).

#### 2.8.3. Cell Membrane Roughness

To obtain the membrane roughness of MCF-7 and SKBR-3 cells, after 24 h and 48 h of treatment with hUMSCs-CM respectively, cells were washed once with PBS and were then fixed in 2.5% glutaraldehyde at room temperature for 15 min followed by an additional washing cycle after the indicated incubation time. Subsequently, cells were dehydrated with 10, 30, 50, 70, 90, and 100% ethanol gradient for 20 min in each step. Atomic force microscopy (AFM) device of the laboratory (Nanoscope IIIa, Veeco, CA, USA) was employed to observe membrane topography and determine the roughness and the 3D profile of the substrates. The surface roughness parameter RMS (round mean square) was calculated in tapping mode on 20 μm × 20 μm area. The cantilevers we used were DNP-S10 probes (Bruker) with 0.6 μm thickness and a spring constant of 0.175 N/m.

### 2.9. Immunofluorescence Assay Prior to and Post Treatment

For 2D surfaces, cells were grown in 12-well plates filled with media and 10% FBS in collagen-coated glass cover slides (40 × 10^3^ cells/well). For 3D scaffolds, cells were seeded upon collagen-coated PCL scaffolds. After 24 h and 48 h incubation of MCF-7 and SKBR-3 with hUMSCs-CM, respectively, cells were fixed with 4% paraformaldehyde when examined 2D surfaces and 3.7% for 3D scaffolds and permeabilized with 0.1% Triton X-100. Cells were then rinsed with wash buffer (1 × PBS containing 0.05% Tween-20). Blocking was performed with 3% bovine serum albumin in phosphate-buffered saline containing 10% FBS for 1 h at RT. After blocking, cells were labeled with TRITC conjugated Phalloidin (1:200; Millipore) for 1 h at RT and then rinsed with wash buffer. Hoechst 33258 (1:4000; Sigma-Aldrich) was used for nuclear counterstaining, and then cells were mounted. Z-stack imaging was conducted on a Leica SP5 TCS equipped with a × 40/1.25NA oil immersion lens. Overall, approximately 200 cells for each case of control and treatment were studied after three repeated experiments and the mean fluorescence intensity of the sum projection was evaluated after background subtraction with the use of the software ImageJ.

### 2.10. Cell Morphology Prior to and Post Treatment

The topography of the scaffolds was investigated using a scanning electron microscope (SEM) (JEOL-JSM 6300). Fiber diameter and surface pore size for the electrospun substrates were calculated using ImageJ software (National Institutes of Health, Bethesda, MD, USA). Cell fixation protocol was relative to AFM protocol. After the final step, samples were coated with gold and examined under SEM with an accelerating voltage of 20 kV.

### 2.11. Statistical Analysis

The results were statistically analyzed using Matlab R2021a. Origin Pro software was used for image plotting. For each case of control and treated over three repeated experiments. ImageJ software was employed for immunofluorescence analysis. All results are expressed as mean ± standard deviation (SD) from at least three independent experiments. Differences between groups and controls were tested by one-way analysis of variance (ANOVA) and considered significant when *p* < 0.05.

## 3. Results

### 3.1. Physicomechanical Propertied of 3D Scaffold

**Figure 2:**
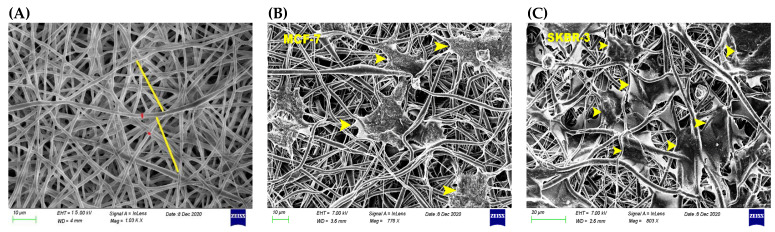
SEM micrograph of 3D PCL scaffolds, yellow and red lines indicate the created pore size and the fiber diameter of PCL scaffolds, respectively (**A**). Cells were seeded on 3D PCL collagen immersed scaffolds and tested for cell viability and morphology during a dynamic condition in the control and post treatment phases. Yellow arrows indicate the way of MCF-7 (**B**) and SKBR-3 (**C**) cells were seeded into 3D scaffolds. Moreover, cancer cells exhibited immunofluorescence assay to estimate the post-treated F-actin morphology.

The 3D fabricated scaffold via the electrospinning method was evaluated with the help of a scanning electron microscope. The microarchitecture of PCL scaffold is illustrated in Figure 2. Randomly distributed fibers were characteristic of a successful electrospun scaffold. The created pores were 24.42 ± 3.01 μm in size, which was close to the diameter of tested cancer cells (Figure 2A). Notably, the average cell diameter of MCF-7 cells was measured 17.46 ± 1.92 μm (Figure 2B), and for SKBR-3 14.08 ± 1.55 μm (Figure 2C) [16]. The fiber diameter of PCL scaffold was found to be 1.26 ± 0.07 μm. The mechanical properties of the 3D scaffold, and more precisely the modulus of elasticity in the linear region of stress–strain curve was measured as 5.79 ± 1.48 MPa. The maximum tensile strength according to the tensile test was found to be 2.08 ± 0.38 MPa, which is in line with results of other studies [18].

### 3.2. Effect of hUMSCs-CM Therapy on Cell Viability after Dynamic Cell Culture

**Figure 3:**
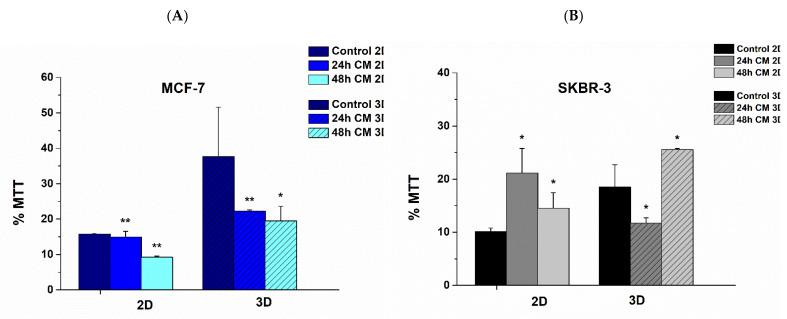
Cell viability of (**A**) MCF-7 and (**B**) SKBR-3 cells in dynamic conditions in both 2D surfaces and 3D PCL scaffolds. Data represents mean values ± SD of three independent experiments, * *p* < 0.05 and ** *p* < 0.01.

Regarding MCF-7 cells in 2D surfaces, cell viability reduced 12% at 24 h and 30% at 48 h post treatment with hUMSCs-CM, respectively. MCF-7 cells reduced their viability by 40 and 45% after 24 h and 48 h of treatment, correspondingly, when cultured on 3D scaffolds in a significant statistic way (Figure 3A).

SKBR-3 cells followed a different trajectory regarding their viability. When cultured on 2D surfaces, cell viability increased 100% after 24 h of treatment with hUMSCs-CM and 50% after 48 h. Considering 3D scaffolds, SKBR-3 cells reduced cell viability 36.8% following through an increase of approximately 100% (Figure 3B). The results indicate that hUMSCs-CM alters the metabolic profile of all cells in both 2D and 3D substrates. Moreover, the therapy seems to correspond mainly to ER+ cells, which are considered less aggressive and invasive than HER2+ regarding cancer progression [19].

### 3.3. Attenuation of Cell Migration Post hUMSCs-CM Treatment

**Figure 4:**
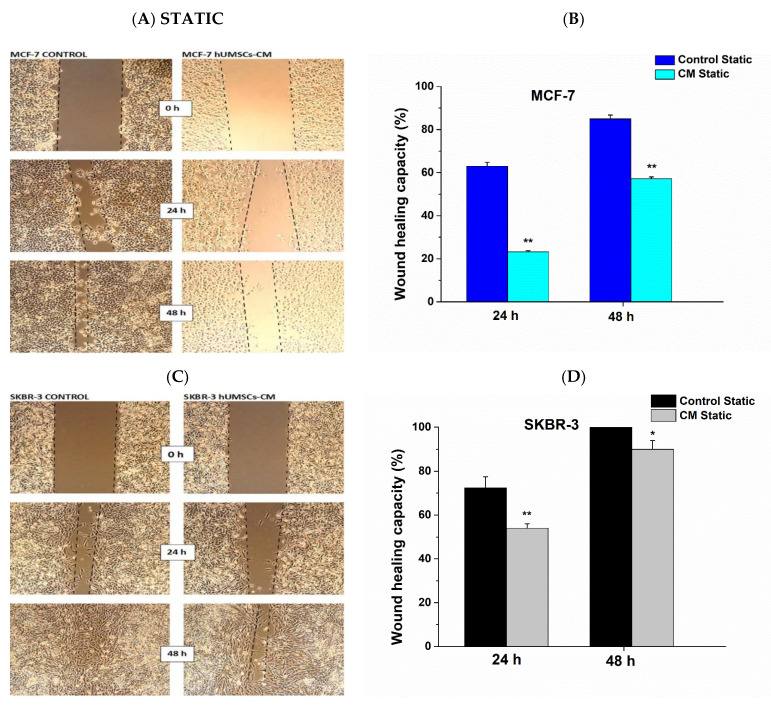
Quantitative analysis of the scratch-wound healing assay after 24 h and 48 h during static conditions. The proliferation of the MCF-7 (**A, B**) and SKBR-3 cells (**C, D**) was examined in the absence (control) and presence of hUMSCs-CM. Data represent mean values ± SD of three independent experiments were performed in both assays, * *p* < 0.05 and ** *p* < 0.01.

The effect of hUMSCs-CM on MCF-7 and SKBR-3 collective cell migration was evaluated using wound healing assays in both static and dynamic conditions. A statistically significant decrease in the ability of cells to migrate was shown in both MCF-7 and SKBR-3 cancer cells. More specifically, in static conditions, the healing capacity of MCF-7 decreased post treatment with hUMSCs-CM 63.5 and 32.9% after 24 h and 48 h correspondingly (Figure 4A,B). Regarding SKBR-3 cancer cells, the healing capacity decreased 25.4 and 10% after 24 h and 48 h of treatment, respectively (Figure 4C,D).

**Figure 5:**
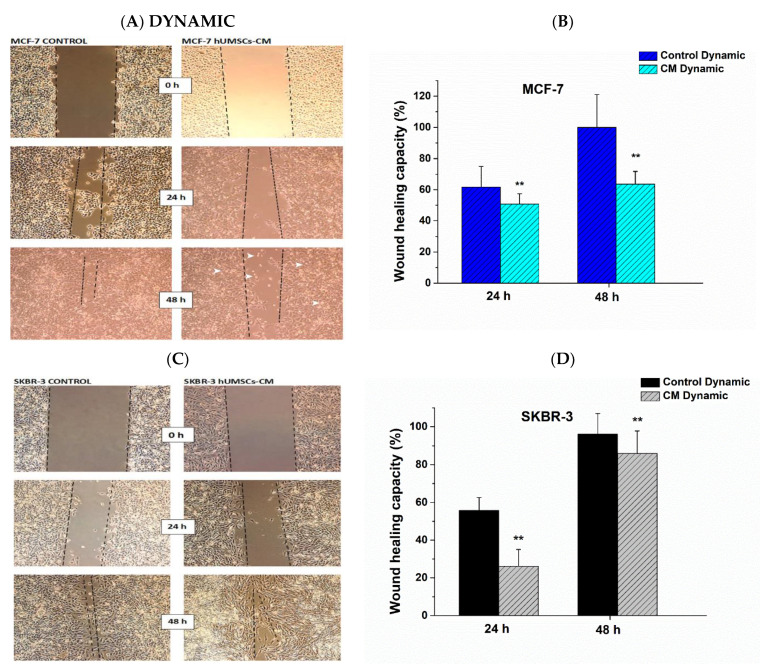
Quantitative analysis of the scratch-wound healing assay after 24 h and 48 h during dynamic conditions. The proliferation of the MCF-7 (**A**,**B**) and SKBR-3 cells (**C**,**D**) was examined in the absence and presence of hUMSCs-CM. Data represent mean values ± SD of three independent experiments were performed in both assays, * *p* < 0.05 and ** *p* < 0.01.

Regarding wound healing after inserting 6-wells into the bioreactor, a statistically significant decrease in the ability of cells to migrate was shown in both cancer cell lines. More specifically, control MCF-7 cells covered 61.76% of the migrated area with cells after 24 h and 99.99% after 48 h, while in the case of treated cells, the relative area covered with cells 50.78 and 63.61% after 24 h and 48 h, respectively (Figure 5A,B). Interestingly, in the case of treated MCF-7 cells, after 24 h and mainly after 48 h a plethora of apoptotic cells were observed (white arrows, Figure 5A). The healing capacity of control SKBR-3 cells was 55.70% after 24 h of inserting in bioreactor and 96% after 48 h; 26.12 and 86% of the migrated cell area was covered by treated cells with hUMSCs-CM after 24 h and 48 h correspondingly (Figure 5C,D).

### 3.4. Alterations in Mechanical Properties Post Treatment with hUMSCs-CM

**Figure 6:**
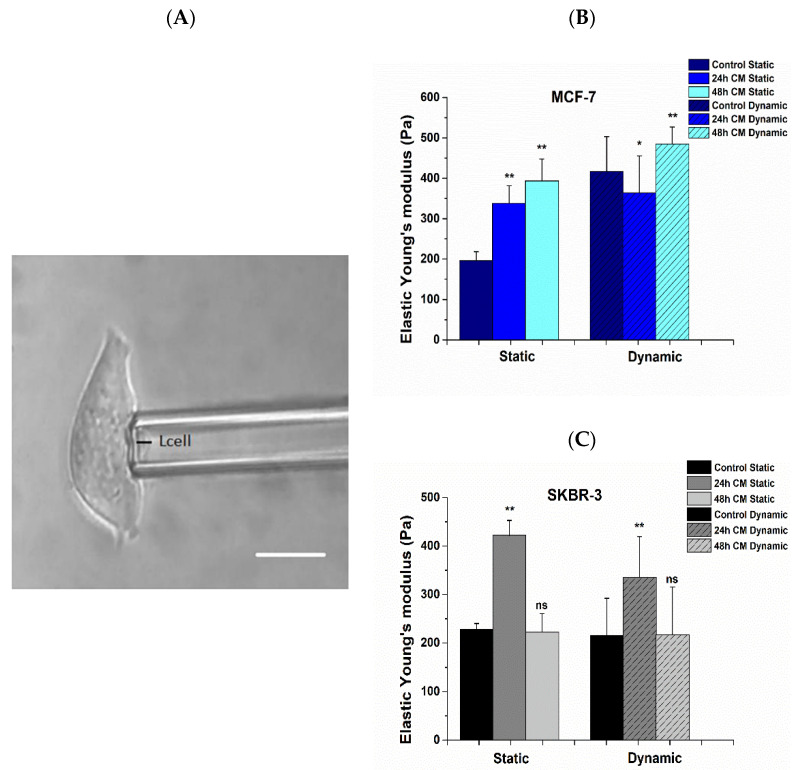
(**A**) Representative image of MCF-7 aspirated cell during micropipette aspiration technique. The cell length (black line) prolongation was recorded under a range of suction pressure (white color scale bar 10 μm, ×20 microscope lens). The Elastic Young’s modulus of adhered cells prior to and post treatment with hUMSCs-CM for a range of applied pressure, 0.05÷340 Pa was determined via Equation (2). (**B**) the increased elastic modulus post treatment in both 24 h and 48 h of static culture and 48 h in dynamic culture indicates a stiff cancer cell with a confined potential for cell motility for MCF-7 cancer cells. In the case of SKBR-3 cells, (**C**) the elastic modulus increases after 24 h of treatment and decreases after 48 h of treatment in both static and dynamic conditions revealing the remaining aggressiveness of this cancer phenotype. Data represents mean values ±SD of three independent experiments, ^ns^ *p* > 0.05, * *p* < 0.05 and ** *p* < 0.01.

Mechanical properties were obtained for each case prior to and post treatment with hUMSCs-CM, and the elastic Young’s modulus was determined after cell aspiration (Figure 6A) via Equation (2). After static cell culture, the elastic Young’s modulus, *E*, of control MCF-7 cells was found to be 196.32 ± 21.68 Pa, while in post-treated cells, the *E* value was increased to 338.09 ± 142.58 Pa and 393.90 ± 53.95 Pa at 24 h and 48 h of treatment respectively (*p* < 0.01) (Figure 6B, Static). In the case of SKBR-3 cells, the elastic Young’s modulus of cells prior to treatment was found to be 277.86 ± 12.57 Pa (Figure 6C, Static). After 24 h the elastic Young’s modulus was increased to 422.04 ± 30.82 Pa (*p* < 0.01) and after 48 h of treatment decreased to 223.0 ± 37.43 Pa (*p* < 0.05). In dynamic conditions, the *E* value was determined for MCF-7 control cells to be 417.13 ± 115.09 Pa following a slow decrease 363.86 ± 155.74 Pa (*p* < 0.05) after 24 h of treatment, resulting in a further increase 484.81 ± 70.45 Pa after 48 h of treatment with hUMSCs-CM (*p* < 0.01) (Figure 6B, Dynamic). Regarding SKBR-3 control cells, the elastic Young’s modulus was found to be 215.20 ± 76.95 Pa with further increase 335.15 ± 84.21 Pa (*p* < 0.01) after 24 h of treatment resulting in 216.52 ± 98.93 Pa post 48 h of treatment, without a statistically significant difference (*p* > 0.05) (Figure 6C, Dynamic). Overall, juxtaposing static and dynamic conditions, the value of elastic Young’s modulus was markedly increased after dynamic culture in the case of MCF-7 cells during all steps of treatment, while statistically significant differences in the elastic Young’s modulus were not observed in SKBR-3 cells regarding control and 48 h post treatment cells.

### 3.5. Effect of hUMSCs-CM on Cell Membrane Roughness

**Figure 7:**
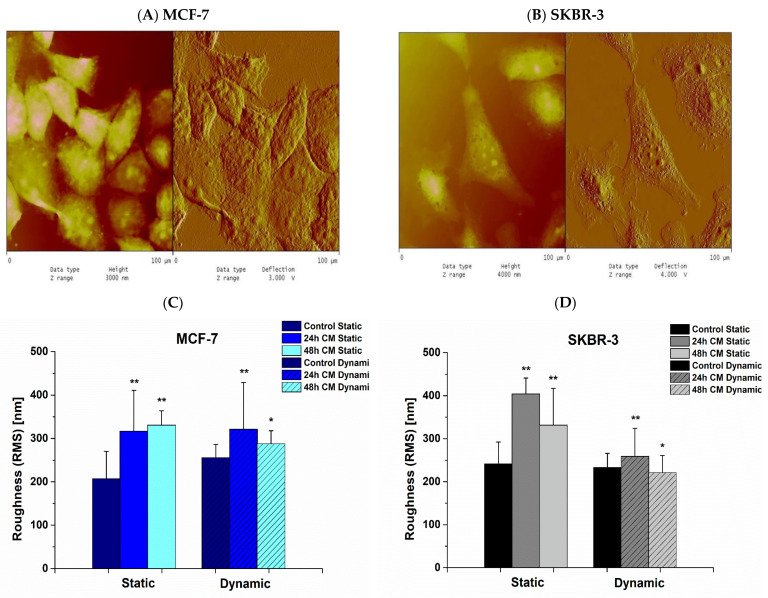
Representative AFM images of MCF-7 (**A**) and SKBR-3 (**B**) cancer cells 48 h post treatment after dynamic conditions. Quantitative AFM analysis displaying roughness of MCF-7 (**C**) and SKBR-3 (**D**) cancer cells’ membrane during 24 h and 48 h of treatment with hUMSCs-CM in both static and dynamic conditions. Data represent mean values ± SD of three independent experiments, * *p* < 0.05 and ** *p* < 0.01.

Cancer cells exhibited AFM in order to obtain the cell membrane roughness in both static and dynamic conditions. In Figure 7, representative images of MCF-7 (Figure 7A) and SKBR-3 (Figure 7B) are depicted undergoing AFM. In static conditions, cell membrane roughness, regarding MCF-7 cells, was found to be 206.98 ± 63.53 nm in control cells while 316.84 ± 93.85 nm and 330.76 ± 33.04 nm after 24 h and 48 h of treatment with hUMSCs-CM, correspondingly (*p* < 0.01) (Figure 7C, Static). In the case of SKBR-3 cells, the membrane roughness was determined to be 241.39 ± 51.03 nm in control cells, 404.02 ± 37.00 after 24 h of treatment, and 331.74 ± 85.28 after 48 h (*p* < 0.01) (Figure 7D, Static). After dynamic conditions cell membrane roughness in MCF-7 cells estimated 255.91 ± 30.45 nm in control, 321.48 ± 107.49 nm post 24 h of treatment (*p* < 0.01) and 288.00 ± 30.13 nm post 48 h of treatment (*p* < 0.05) (Figure 7C, Dynamic). SKBR-3 cell membrane roughness of control cells was determined to be 233.08 ± 32.96 nm, and after 24 h and 48 h of treatment 259.09 ± 64.99 nm (*p* < 0.01) and 221.58 ± 39.17 nm, respectively (*p* < 0.05) (Figure 7D, Dynamic). Overall, juxtaposing static and dynamic conditions, only slight differences were observed in cell membrane roughness of both cancer cell lines when cells exhibited dynamic conditions. In static cell culture, cell membrane roughness was steadily increased.

### 3.6. Effect of hUMSCs-CM Treatment on F-actin Morphology and Mean Intensity

**Figure 8:**
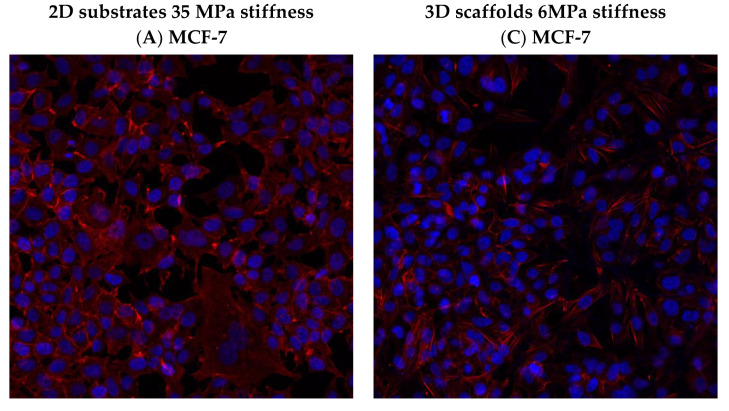

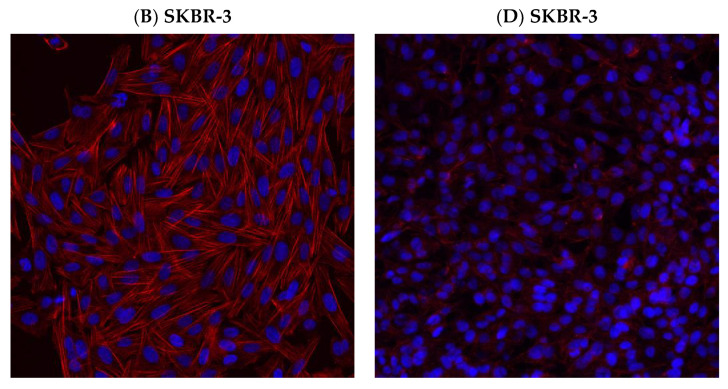
Representative image of F-actin morphology (red) and nuclei (blue) of MCF-7 and SKBR-3 control cells seeded upon 2D (**A**,**B**) and 3D scaffolds (**C**,**D)** with different stiffness. SKBR-3 morphology was markedly altered when cells cultured upon 3D PCL scaffolds. Analytical images of 24 h and 48 h post treatment are depicted in Supplementary Figures S1–S4.

Alterations in cytoskeletal morphologies of F-actin were detected in post treated with hUMSCs-CM cancer cells in both 2D surfaces and 3D scaffolds, and are depicted in Supplementary Figures S1–S4, while Figure 8 depicts solely control cells with F-actin and nucleus staining. In particular, upon 2D collagen-coated surfaces, in the case of control MCF-7 cells, F-actin fibers were detected all over the extended cell membrane and specifically at the leading edge indicating the increased migratory potential (Figure 8A). On the contrary, after 24 h of treatment and major 48 h of treatment, F-actin was perinuclear accumulated and less flattened compared to untreated cells (Supplementary Figure S1). Moreover, the F-actin stress fibers were evenly distributed in the cell body of treated MCF-7 cells, a condition that corroborates with the limited potential for migration.

The morphology of SKBR-3 cells on 2D surfaces was quite different. F-actin stress fibers were fully polymerized, and the remarkable polygonal cell architecture of untreated cells is widely considered as the morphology related to enhanced migration and invasion (Figure 8B) [20]. On the contrary, after 24 h of treatment, the intense F-actin fibers were significantly depolymerized and confined, hence revealing a more spherical-like shape, implying thus the limited cell movement (Supplementary Figure S2). This finding is in line with the stiffness of 24 h post-treated SKBR-3 cells where elastic Young’s modulus was significantly increased. Moreover, after 48 h of treatment with hUMSCs-CM, F-actin in SKBR-3 cells started to further polymerize and regain the polygonal morphology by shaping protrusions and pseudopods (Supplementary Figure S2). This finding is also in line with the elastic Young’s modulus of cells in these conditions, which dramatically decreased, implying a softer and more kinetic cell [16].

Regarding F-actin shape distribution in MCF-7 cells, cultured on 3D scaffolds, no significant changes in the morphology of stress fibers were detected. MCF-7 cells were maintained their spherical-like shape, though the seeding density was higher in untreated cells (Figure 8C and Figure S3). Considering SKBR-3 cells, the cell morphology altered dramatically compared to 2D conditions. F-actin noted a significant reorganization when polymerized in 3D scaffolds, which was also polygonal but “smoother” (with limited extended stress fibers) than in 2D surfaces (Figure 8D and Figure S4).

**Figure 9:**
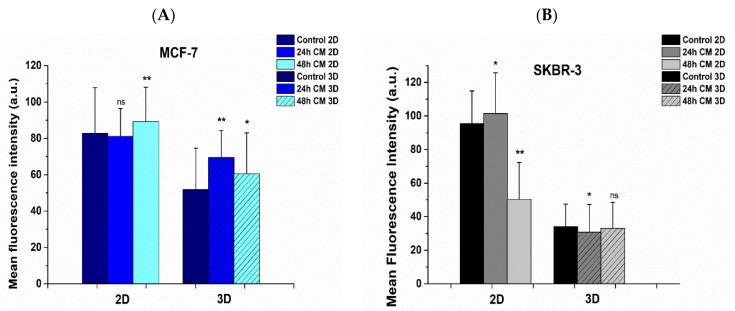
Quantitative confocal microscopy analysis via ImageJ displaying mean fluorescence intensity. MCF-7 (**A**) and SKBR-3 (**B**) cancer cells were incubated with hUMSCs-CM at 24 h and 48 h, respectively. F-actin was stained with conjugated phalloidin and nucleus with Hoechst. Quantification of F-actin mean intensity yielded differences in actin staining that were consistent with the trends observed in the cell stiffness measurements. Data represent mean values ±SD of mean immunofluorescence intensity of more than three independent experiments are represented, ^ns^ *p* > 0.05, * *p* < 0.05. ** *p* < 0.01.

The mean intensity of F-actin was increased in MCF-7 post-treated cells in both 2D substrates and 3D scaffolds, implying firmly attached cells in the ECM (Figure 9A). However, the F-actin mean intensity decreased in SKBR-3 cells post treatment, in the case of 2D substrates, indicating the remaining potential for cell movement (Figure 9B).

Regarding the mean intensity distribution of F-actin on 3D scaffolds in both breast cancer cell lines and in all cases of control and treated, overall, the mean intensity was decreased juxtaposing 2D surfaces (Figure 9A,B). This finding could be interpreted with the fact that cell membrane on 2D surfaces tends to spread wider than those in scaffolds, which spread in three dimensions, thus increasing the average intensity by distributing a given signal over a greater area. Moreover, the 2D substrate stiffness was 35 MPa and significantly greater than 3D scaffolds of 6 MPa stiffness, encouraging thus the maximum polymerization of F-actin fibers and therefore the maximum extension of the cell membrane.

## 4. Discussion

In the latest decades, several efforts have been dedicated to deciphering biomechanical properties of cancer cells as promising biomarkers for cancer prognosis and diagnosis. Biomechanics of cancer cells alters during the process of metastasis. Notably, evading cancer cells from the initial tumor microenvironment become more deformable in order to invade surrounding tissues. Therefore, the cell elasticity is constantly altered as cancer cells undertake dramatically mechanical stresses and respond with cell membrane deformation. Furthermore, cell membrane roughness as a significant cytological parameter, contributing to a myriad of cellular mechanisms, and specifically in cell adhesion and motility, also changes during metastatic cascade [21,22].

However, the effect of chemotherapeutic drugs and other anti-cancer therapies upon cancer cells biomechanics is still ongoing [23,24]. In our previous work, we addressed that specific anti-tumor agents altered the biomechanics of MCF-7 and SKBR-3 cells with different cancer phenotypes expressing ER and HER2 correspondingly [16]. In this current study, we further sought to elucidate the stem cell-like therapeutic potency. Particularly, conditioned medium was derived from hUMSCs on aforementioned cancer cell lines and the alteration in cell viability, mechanical properties, cell membrane roughness and morphology, and migratory potential during static and dynamic culture conditions were determined.

Moreover, the cancer cell viability was evaluated prior to and post treatment with hUMSCs-CM upon 2D surfaces and 3D scaffolds. 3D scaffolds were utilized in this study to create a more appropriately engineered cell culture environment and improve the physiological equivalence of in vitro experiments [25]. The cancer cells’ microenvironment inside electrospun 3D structures satisfactorily mimics the specific microarchitecture of living tissues [26]. Cancer cell viability was also assessed after static and dynamic cell culture. Interestingly, our results showed reduced post treatment cell viability in static and dynamic conditions of ER+ breast cancer cells. However, overall cell viability increased post treatment in the case of HER2+ cancer cells. In parallel, the reduced post treatment migrated cell area of MCF-7 cells in both static and dynamic conditions indicates a limited tension of dissemination, cell movement, and hence metastasis.

To further elucidate the underlying mechanisms of alterations in biomechanics of cancer cells undergoing therapy with stem cells medium, the cell stiffness and cell membrane roughness of both treated and control cancer cells were determined [27]. A plethora of studies addressed that normal cells are stiffer than cancer cells, hence, recent studies have focused on elucidating differences between treated and non-treated cancer cells [16,17,28,29]. The importance of understanding the mechanical response of cytoskeletal networks is crucial in deciphering the cell membrane stiffness, which is intertwined with F-actin, the cytoskeletal protein which provides cells with mechanical support [30,31]. In the case of ER+ cancer cells, a significant trend was revealed; the increased post treatment elastic Young’s modulus in both static and dynamic conditions during 24 h and 48 h of treatment with hUMSCs-CM outlines the increased cell stiffness, which indicates a limited potential in cell migration and restricted cell motility. These results are in line with our previous study addressing that alteration in the mechanotransduction cascade of post-treated with anti-tumor agents cells that became stiffer had also a limited tendency for cell motility and restricted potential in migration [16]. Furthermore, the increased post treatment cell membrane roughness of MCF-7 cells in combination with F-actin reorganization in both static and dynamic conditions contributes also to a stiffer cell with limited cell motility as previous studies established [32,33]. Similar morphology of MCF-7 cells with reduced membrane ruffles, pseudopodia, and lamellipodia was detected in the study of Flamini et al., post treatment with retinoic acid, indicating a restricted migratory phenotype [34]. On the contrary, HER2+ cancer cells exhibited a different behavior. Particularly, SKBR-3 cells showed a remarkable increase in both elastic Young’s modulus and cell membrane roughness after 24 h of treatment following by a dramatic decrease after 48 h of treatment during static and dynamic conditions. This behavior could be interpreted considering the more aggressive profile of SKBR-3 cell line as HER2+ cells with the intrinsic capacity to induce pseudopods and protrusions, thus evading the specific therapy by regaining their initial (prior treatment) deformable cell membrane, and hence favoring cell motility and migration [35,36].

Our findings regarding the treatment effect of hUMSCs-CM upon MCF-7 cells are in line with those of Yuan Yin et al., which addressed that hUMSCs-CM induced apoptosis of lung cancer cells by downregulating pro-caspase-7 and Bcl-2, indicating the activation of intrinsic and extrinsic apoptosis pathways [10]. Moreover, our results regarding attenuation in cell migration and alterations in the biomechanics of MCF-7 cells in a way that restricts cell motility were corroborated via the biological pathway from the study of Qiao et al., revealing that β-catenin was down-regulated in MCF-7 cells treated with conditioned medium from human mesenchymal stem cells (hMSCs), and specifically, Dkk-1 inhibitors secreted in the conditioned media from hMSCs played a key role resulting in depression of Wnt signaling [11]. This biological pathway could interpret our results regarding attenuation in cell migration and alterations in the biomechanics of ER+ MCF-7 cells in a way that restricts cell motility.

On the other hand, the more aggressive HER2+ SKBR-3 cells followed a different biological response post treatment with hUMSCs-CM. In the case of aggressive breast cancer cells, namely triple-negative cancer, the knockdown of β-cantenin induces a reverse-effect following the reduction of E-cadherin expression levels leading to increased cell motility and epithelial to mesenchymal transition as the study of Cai et al. showed [37]. Hence, this biological pathway may affect HER2+ cells of our study, which are morphologically close to triple-negative cells with induced cell protrusions and pseudopods.

According to previous studies, rat-tail collagen I stiffness was 5.1 GPa [38,39], whereas the stiffness of PCL 3D scaffolds was estimated in this current study at approximately 6 MPa. The increase in 2D substrate stiffness was positively related to the extension of cells by the determination of cell surface area and polarity index. Moreover, it is widely accepted that the increased cellular stiffness weakens the ability of the cytoskeleton to undergo dynamic intrinsic changes that favors migration and invasion processes [40]. Overall, juxtaposing static and dynamic conditions regarding cell viability, the seeded density was enhanced in the dynamic microenvironment in the case of control cells, while cell viability decreased in ER+ post-treated cells. Moreover, cell elasticity was affected after dynamic cell culture in both ER+ and HER2+ breast cancer cells, while cell membrane roughness increased only in ER+ cells after exhibiting dynamic conditions. The therapy with hUMCSs-CM enhances the acquisition of novel cellular behavior, including restrictions in cell motility during migration, increased morphological and cytoskeletal reorganizations, and alterations in cell biomechanics.

## 5. Conclusions

This study provides new insights and encourages the development of modalities for screening new therapeutic anti-cancer drugs regarding stem cell therapy. Concluding, we suggest that hUMSCs-CM as a treatment can be satisfactorily applied in the case of ER+ MCF-7 cancer cells. Particularly, the dynamic microenvironment of 3D ECM scaffolds and the bioreactor was satisfactorily applied to MCF-7 cancer cells, where the cell viability reduced markedly post treatment. Moreover, the stiffness of MCF-7 cells was significantly increased in both static and dynamic conditions, indicating a more rigid cytoskeletal with restricted cell movement. These findings were in line with the restricted cell migration post treatment in both static and dynamic conditions and were corroborated with the confined formation of lamellipodia and membrane ruffles. In the case of SKBR-3 HER2+ cells, we postulate that the treatment was not efficient in achieving a confined cell viability and motility of cancer cells. Overall, the cell viability of SKBR-3 cells was significantly increased, indicating a proliferative inclination and a more aggressive phenotype. Moreover, the decreased post treatment stiffness in both static and dynamic cell conditions of SKBR-3 cells indicate a deformable cell with a potency for cell motility and, therefore, migration. In line with this evidence was the F-actin morphological profile of SKBR-3 cells, which re-polymerized 48 h post treatment, hence regaining the extended stress fibers, resulting in enhanced probability for cell movement and migration. Future studies upon stem cell therapy treatment on cancer cells and circulatory tumor cells (CTCs) derived from patients will elucidate further and integrate the contribution of this work [41,42].

## Data Availability

Generated and analyzed data of this study are included in this published article and its supplementary material files.

## Supplementary Materials

The following are available online at https://www.mdpi.com/article/10.3390/bioengineering8110189/s1, Figure S1: F-actin morphological changes in MCF-7 cells of control and treated with hUMSCs-CM at 24 h and 48 h, upon collagen coated type I substrates. Interestingly, post 48 h of treatment F-actin is perinuclear co-located indicating a restricted cell motility, Figure S2: F-actin distribution in SKBR-3 cells seeded on collagen coated type I 2D substrates. In control cells F-actin stress fibers revealed fully extended whereas post 24 h of treatment F-actin depolymerized, following a further polymerization after 48 h of treatment, Figure S3: F-actin morphology of MCF-7 cells upon PCL 3D scaffolds. Cellular morphology was not significantly changed; however, the cell density of control cells was higher than post treated cells, Figure S4: F-actin distribution of SKBR-3 cells upon PCL 3D scaffolds. Cell morphology was altered significantly post 24 h of treatment with hUMSCs where F-actin located perinuclear giving a spherical-like shape to cells. Post 48 h of treatment, F-actin seems to re-polymerized with enhanced stress fibers.
