# Supplementary material for: Engineering Breast Cancer Cells and hUMSCs Microenvironment in 2D and 3D Scaffolds: A Mechanical Study Approach of Stem Cells in Anticancer Therapy
Source: Bioengineering (Basel). 2021 Nov 20;8(11):189. doi: 10.3390/bioengineering8110189 (PMC8615245; doi:10.3390/bioengineering8110189)
Supplement: Supplementary file 1 [file bioengineering-08-00189-s001.zip › bioengineering-1463744-supplementary.pdf]

MCF7 2D substrates

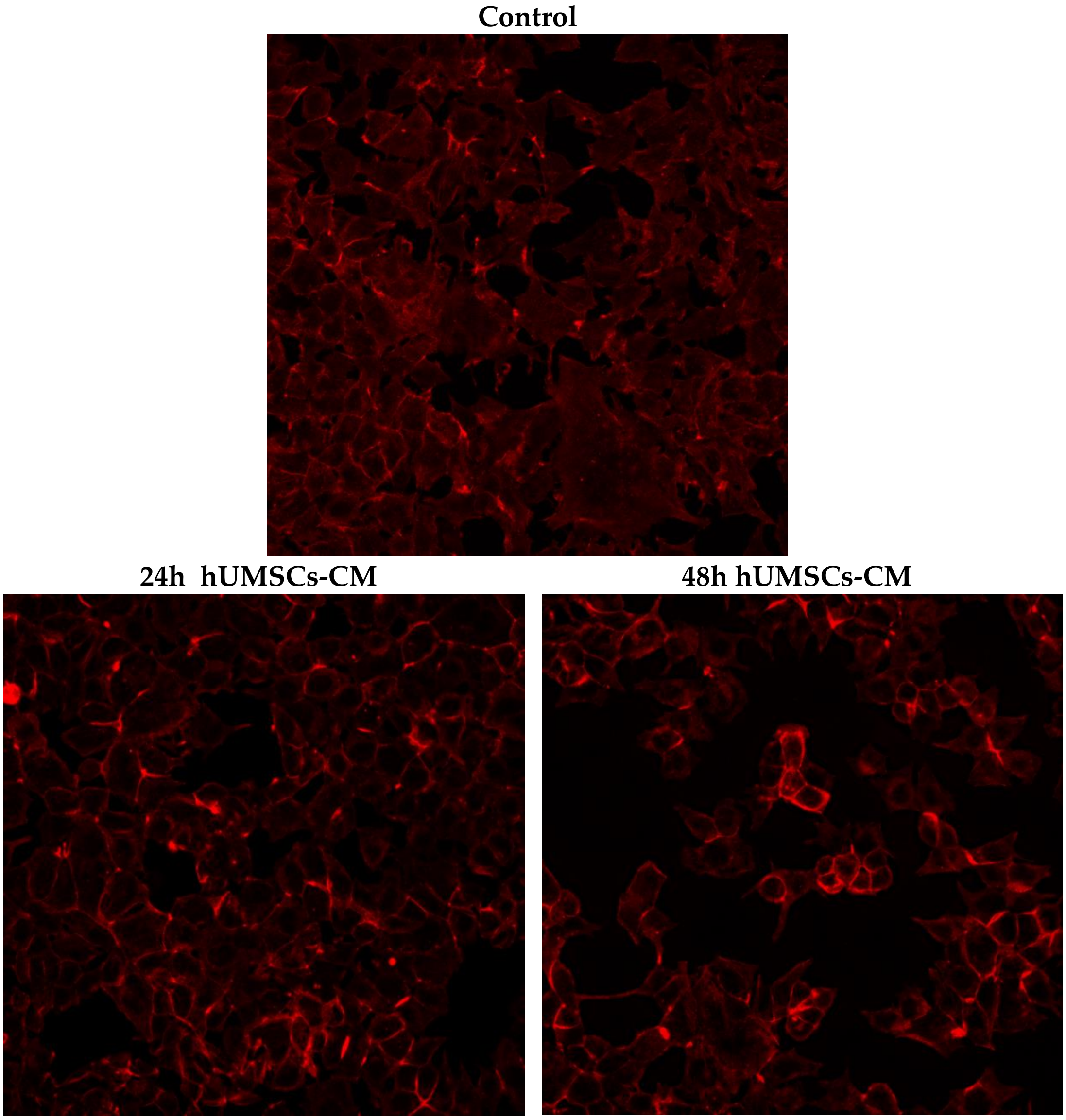

Supplementary Figure S1. F-actin morphological changes in MCF-7 cells of control and treated with hUMSCs-CM at 24h and 48h, upon collagen coated type I substrates. Interestingly, post 48h of treatment F-actin is perinuclear co-located indicating a restricted cell motility

SKBR3 2D substrates

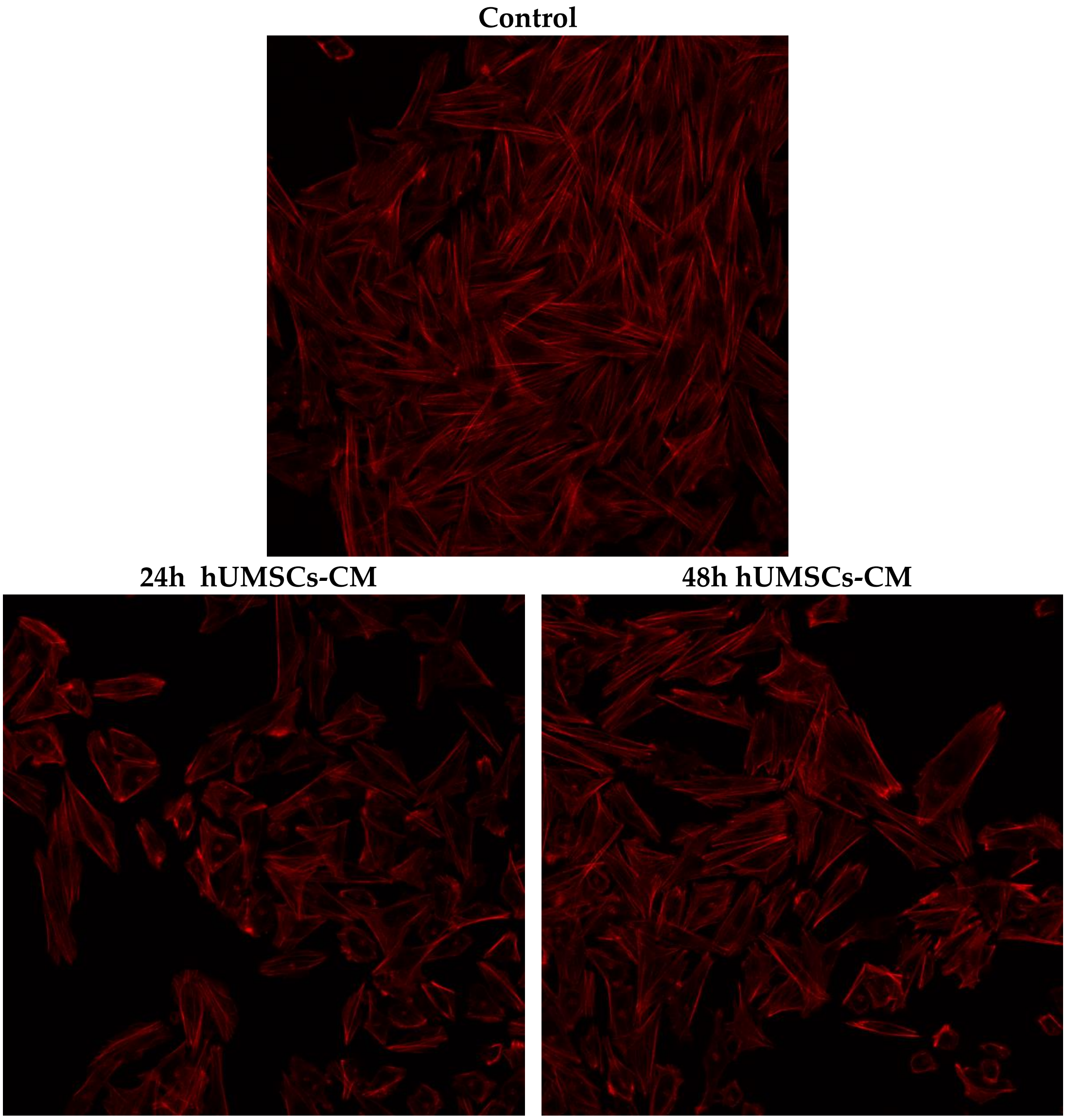

Supplementary Figure S2. F-actin distribution in SKBR-3 cells seeded on collagen coated type I 2D substrates. In control cells F-actin stress fibers revealed fully extended whereas post 24h of treatment F-actin depolymerized, following a further polymerization after 48h of treatment.

MCF7 3D scaffolds

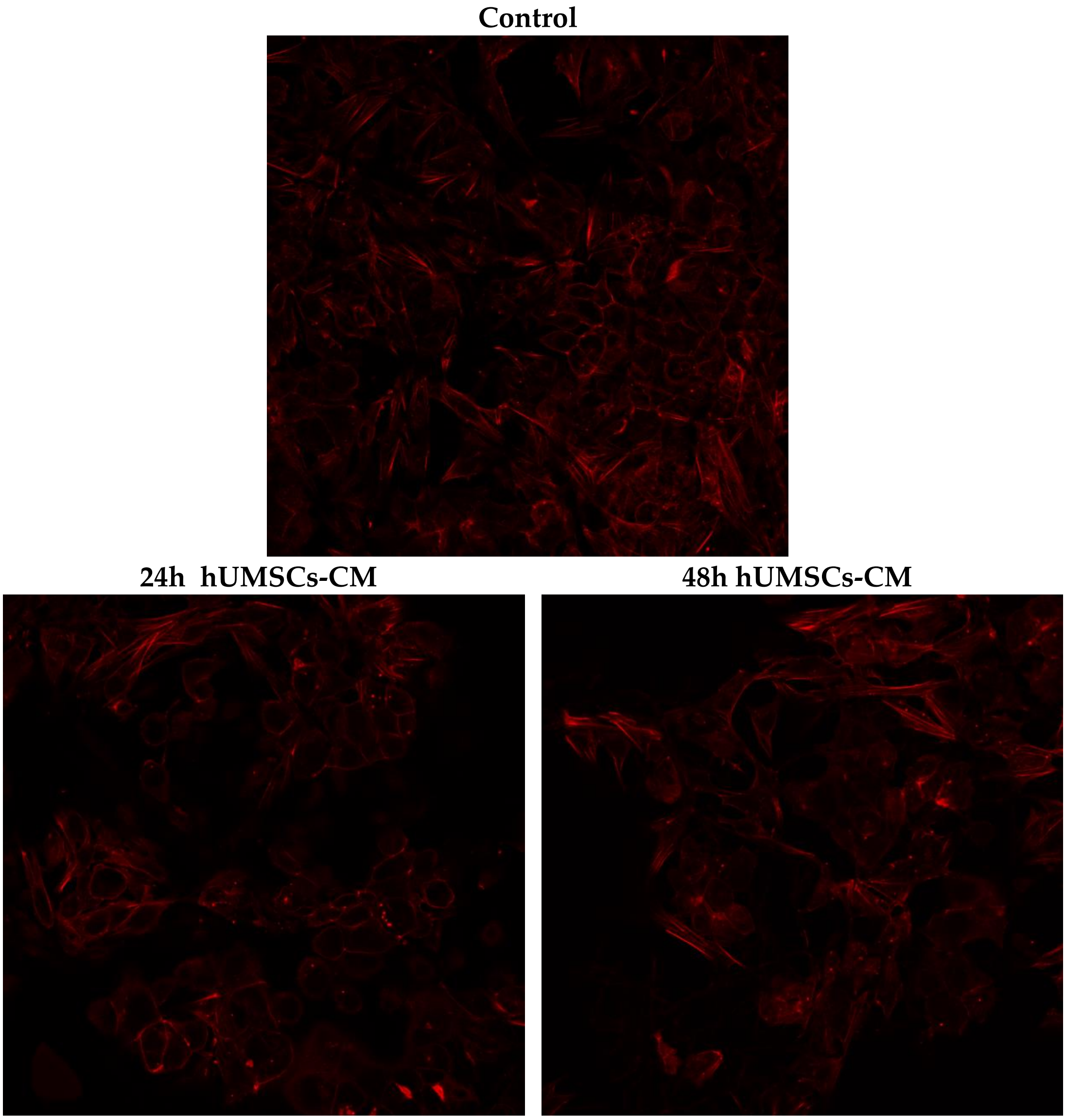

Supplementary Figure S3. F-actin morphology of MCF-7 cells upon PCL 3D scaffolds. Cellular morphology was not significantly changed; however, the cell density of control cells was higher than post treated cells.

SKBR3 3D scaffolds

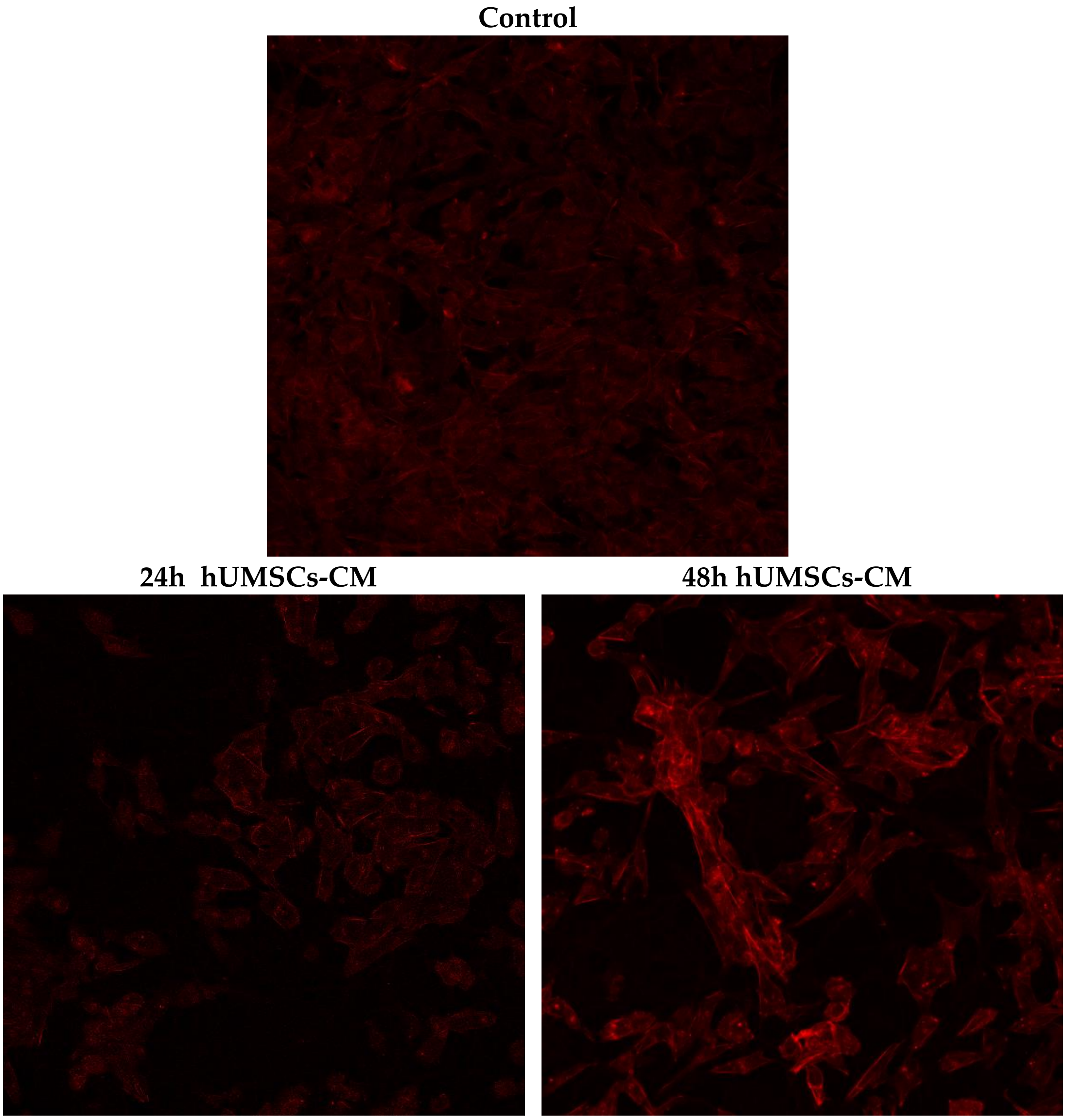

Supplementary Figure S4. F-actin distribution of SKBR-3 cells upon PCL 3D scaffolds. Cell morphology was altered significantly post 24 h of treatment with hUMSCs where F-actin located perinuclear giving a spherical-like shape to cells. Post 48 h of treatment, F-actin seems to re-polymerized with enhanced stress fibers.
